# Longitudinal and Cross-Sectional Sex Differences in Sleep Health Across Middle and Older Adulthood

**DOI:** 10.1093/geroni/igaf122.488

**Published:** 2025-12-31

**Authors:** Kris Calfee, Soomi Lee

**Affiliations:** The Pennsylvania State University, University Park, Pennsylvania, United States; The Pennsylvania State University, University Park, Pennsylvania, United States

## Abstract

Differences in sleep between males and females have been well-documented; however, this is most often examined using a single assessment of one or limited sleep variables. The current study used the Ru-SATED sleep health framework to examine potential differences in multidimensional sleep health in a sample of 434 adults (Mage=54) from the Midlife in the United States study. A subsample of N = 166 was also used to examine change in sleep health over approximately 10 years. Sleep dimensions of regularity, timing, efficiency, and duration were measured across 7 days using Actigraphy, while satisfaction and alertness were measured by sleep diary during the same 7 days. Each dimension was dichotomized (suboptimal vs. optimal) and summed to create a composite score of sleep health. Cross-sectional analyses indicated that men had more suboptimal overall sleep health compared to women. Specifically, men had less regular sleep, lower evening alertness, lower sleep efficiency, and shorter sleep duration compared to women after controlling for sociodemographic characteristics and chronic conditions. Conversely, men were more likely to have optimal sleep timing, and there were no sex differences in sleep quality. Longitudinally, overall declines in the sleep health composite and the majority of individual domains from baseline to follow-up did not differ by sex, except that sleep duration shortened more for men. These findings indicate that men and women may experience similar age-related declines in sleep health, although men have more sleep health problems at baseline and their sleep duration becomes shorter over time.

